# Plant cell wall hydrolysis process reveals structure–activity relationships

**DOI:** 10.1186/s13007-020-00691-5

**Published:** 2020-11-03

**Authors:** Yanan Zhang, Shengnan Xu, Fan Ji, Yubing Hu, Zhongwei Gu, Bingqian Xu

**Affiliations:** 1 College of Materials Science and Engineering, Nanjing Tech University, Nanjing, 210016 China; 2 National Special Superfine Powder Engineering Research Center of China, Nanjing University of Science & Technology, Nanjing, 210014 China; 3 Single Molecule Study Laboratory, College of Engineering and Nanoscale Science and Engineering Center, University of Georgia, Athens, GA 30602 USA

**Keywords:** *Populus*, Mutant plant cell wall, Biomass degradation, Carbohydrate active enzyme, AFM imaging, Real-time

## Abstract

**Background:**

Recent interest in *Populus* as a source of renewable energy, combined with its numerous available pretreatment methods, has enabled further research on structural modification and hydrolysis. To improve the biodegradation efficiency of biomass, a better understanding of the relationship between its macroscopic structures and enzymatic process is important.

**Results:**

This study investigated mutant cell wall structures compared with wild type on a molecular level. Furthermore, a novel insight into the structural dynamics occurring on mutant biomass was assessed in situ and in real time by functional Atomic Force Microscopy (AFM) imaging. High-resolution AFM images confirmed that genetic pretreatment effectively inhibited the production of irregular lignin. The average roughness values of the wild type are 78, 60, and 30 nm which are much higher than that of the mutant cell wall, approximately 10 nm. It is shown that the action of endoglucanases would expose pure crystalline cellulose with more cracks for easier hydrolysis by cellobiohydrolase I (CBHI). Throughout the entire CBHI hydrolytic process, when the average roughness exceeded 3 nm, the hydrolysis mode consisted of a peeling action.

**Conclusion:**

Functional AFM imaging is helpful for biomass structural characterization. In addition, the visualization of the enzymatic hydrolysis process will be useful to explore the cell wall structure–activity relationships.

## Background

Biomass energy as a renewable carbon source helps to alleviate global climate change by reducing environmental pollution on a large scale. *Populus*, which is an abundant and cheap resource, is widely used [1]. Their native biomass has a complex heterogeneous and hierarchical structure, composed of hemicellulose, lignin, cellulose, and other polysaccharides. One of the best strategies to convert biomass into sugars is enzymatic degradation because it causes less pollution and has a low energy requirement [2]. The required enzymes are generally obtained via industrial fermentation of the important fungus, *Trichoderma reesei* [3]. Endoglucanases Cel7B (EG I) and exoglucanase Cel7A (TrCBHI) prepared from *T*. *reesei* can effectively hydrolyze biomass through a synergistic effect [4]. EG can remove disordered cellulose chains by endo activity. CBHI, which is also known as the “processive” cellulase, appears to catalyze most bond-cleavages in the saccharification of crystalline cellulose [5]. Unfortunately, the crystalline nature of cellulose and the embedded connection with lignin in biomass leads to poor accessibility and weak degradation of cellulose during biomass processing [6]. Non-polysaccharide aromatic polymer lignin, poses significant resistance to microbial and enzymatic deconstruction [7]. Therefore, a selective and efficient pretreatment system and a hydrolysis process to make cellulose more accessible for conversion, are necessary to achieve efficient biomass utilization.

To improve enzyme accessibility of wild type biomass and increase yields of fermentable sugars, different pretreatment technologies, including physical, chemical, physio-chemical, and biological approaches, need to be initiated prior to enzymatic hydrolysis [8]. As reported, lignin cross linking and cellulose crystallinity are major barriers that severely increase the cost of biomass conversion [9]. Recent advances in plant genetic engineering to increase cellulose content, while decreasing cellulose crystallinity and lignin contents, and produce protein modules and hydrolases that disrupt the plant cell wall have been extensively studied [10]. Until now, genetic engineering of woody plant species and food crops has been well established [11–13]. Genetic engineering has achieved remarkable enhancement of ethanol yield and throughput rate, but the mechanism has not been studied sufficiently [14]. A fundamental understanding of pretreatment technology, composition of biomass substrate, and the relationship between the composition of biomass and pretreatment would significantly help to identify an improved pretreatment method. Fourier Transform Infrared (FTIR)、 Nuclear Magnetic Resonance (NMR) and Scanning Electron Microscopy (SEM) have been widely used to characterize the effects of pretreatment and enzymatic hydrolysis on the microstructure of biomass [15, 16]. During processing, changes occurring in cellulose crystalline and aggregate structure have been proved to be of great importance for the properties of the resulting biomass [17]. In this rearrangement, lignin and hemicellulose have also been shown to play a much more central role [18]. These are the reasons why a more precise characterization of the arrangement of different constituents is necessary.

To improve the biodegradation efficiency of biomass, a better understanding of the relationships between its macroscopic structures and enzymatic processes is important. However, with regard to the microscopic level, to understand how the different constitution arrangements contribute to the hydrolysis efficiency and how these interactions are affected by pretreating, vital information is still missing. Visualization techniques have been utilized to represent the detailed information of accessibility and digestibility of cellulase to different biomasses [19, 20]. An overview of biomass visualization technique is presented in Additional file 1: Table S1. He et al. investigated diluted-acid pretreated rice straw hydrolysis in situ using green fluorescence protein (GFP); however, they reported that the binding and hydrolysis efficiency of enzymes might be affected by the modified GFP [21]. Dong et al. applied confocal laser scanning microscopy coupled with fluorescent-labeling techniques to visualize the spatial changes for the accessibility and digestibility of cellulase to the alkali-pretreated biomass [22]. To improve the performance of cellulases, the mechanism of action of these enzymes must be understood at the molecular level. In general, atomic force microscopy (AFM) is a more comprehensive method that provides high quality images of the cellulose substrate with high resolution and allows real-time hydrolysis to be observed in situ [23]. Quirk et al. demonstrated the tremendous potential of AFM for studying the mechanism of enzymatic hydrolysis of cellulose [24]. High-speed atomic force microscopy (HS-AFM) has also been employed to investigate the action of enzymes on crystalline cellulose, thus enabling the dynamic visualization of structural changes [25]. Cell wall hydrolases have a complex molecular architecture consisting of catalytic modules and substrate-targeting carbohydrate-binding modules (CBMs) [26]. CBM binding to cellulose, the very first step of the enzymatic hydrolysis of cellulose, is one of the most significant carbohydrate–protein interactions. Because of the specific interaction with crystalline cellulose, CBM molecules (derived from *Clostridium thermocellum* Scaffoldin CipA) have been chosen as a probe to specifically recognize and map the crystalline cellulose distribution [27]. According to our previous research, AFM topography and recognition imaging can be successfully applied to investigate the complete enzymatic hydrolysis of pretreated cellulose substrates in situ and in real time [28]. The **s**chematic of AFM recognition imaging is presented in Additional file 1: Fig. S1. Through the single molecule study of the complete hydrolytic process, the hydrolytic mode of one pure cellulose microfibril can be obtained.

This study presents an effort to analyze various factors of importance for the understanding of the relationship between the ultrastructure of biomass constituents and their hydrolysis properties. The structures of both wild type and mutant cell walls were explored by high-resolution AFM topography imaging. Furthermore, to better understand the enzymatic digestion process of the mutant cell wall, functional AFM imaging was used to visualize the enzymatic hydrolysis process in situ and in real time. This work will aid investigations of biomass pretreatment and its structural characterization. In addition, the visualization results of the enzymatic hydrolysis process of pretreated biomass could be used as guidance to explore biomass processing and large-scale biofuel production.

## Materials and methods

### AFM tip functionalization

The AFM tips (CS-25 silicon, 0.1 N/m) were purchased from Nanoscience Instruments, Phoenix, AZ, USA. The recombinant CBM 3a (translated amino acid sequence: MGVSGNLKVEFYNSNPSDTTNSINPQFKVTNTGSSAIDLSKLTLRYYYTVDGQKDQTFWCDHAAIIGSNGSYNGITSNVKGTFVKMSSSTNNADTYLEISFTGGTLEPGAHVQIQGRFAKNDWSNYTQSNDYSFKSASQFVEWDQVTAYLNGVLVWGKEPGLEHHHHHH) was provided by the Complex Carbohydrate Research Center, University of Georgia, Athens, GA, USA. The preparation of recombinant CBM 3a and CBM 3a-AFM tip functionalization procedure has been described previously [28]. A Ni–NTA-His system was used to provide accurate orientation of biomolecules. The proper binding between CBM 3a molecules and crystalline cellulose surface was achieved using PEG2000 linker (Nanocs Inc., NY, USA).

### Hydrolysis experiment strategy and AFM data collection

Mutant biomass (*Populus*), in which the expression of a lignin biosynthetic pathway gene 4CL has been downregulated, and wild type biomass, were provided by the CJ’Lab of the department of genetics at the University of Georgia [29]. Endoglucanases (Lot: SLBK0939), cellobiohydrolase I (Lot: SLBF4539), *β*-Glucosidases (Lot: SLBF836ZV) enzyme solution and all chemicals were purchased from Sigma-Aldrich Company. Tris–Cl buffer was used to dilute the enzyme solution to a concentration of 0.0001 U. Stem sections of 40 *μ*m thickness were cut by microtome in the Electron Microscopy Lab (Department of Plant Biology, University of Georgia). 4 mg of microtomed *Populus* slice (wild type and mutant) was fixed onto a cleaned glass surface at 50 °C for 8 h to ensure that the substrate remains stable in solution during the entire experimental period. Then, the glass chip was fixed onto an AFM liquid cell and filled with 0.4 mL Tris–Cl buffer (10 mM Tris–Cl and 150 mM NaCl, pH = 7.5). To add enzyme solutions during the in situ imaging process, the AFM liquid cell was equipped with a capillary port (Φ0.3 mm) on one side. All imaging trials were conducted at 28 °C. AFM was installed in an acoustic and vibration isolation chamber (PicoPlus Isolution Chamber) to ensure absolute stability. During each trial, one target sample area was chosen and scanned for at least 20 min before injecting the enzyme solution. After obtaining one set of high-resolution AFM topography, amplitude, and recognition images, the enzyme solution (50 mM, pH 4.8, sodium acetate buffer) was gently and carefully injected into the liquid cell to ensure minimum interference during the injection process. Then, the hydrolysis process was monitored over the next 7.5 h until the reaction reached equilibrium. All AFM images were taken in the non-contact, Top magnetic AC (TopMAC) mode under the control of Pico TREC (Model N9610A, Agilent Technologies, Santa Clara, CA, USA) with CS-10 silicon AFM tips (0.1 N/m). Each AFM image was obtained at a scanning speed of 6 μm/s. Other scanning parameters were optimized for each acquisition.

## Results and discussion

### Surface mapping of wild type and mutant plant cell walls of *Populus*

**Fig. 1:**
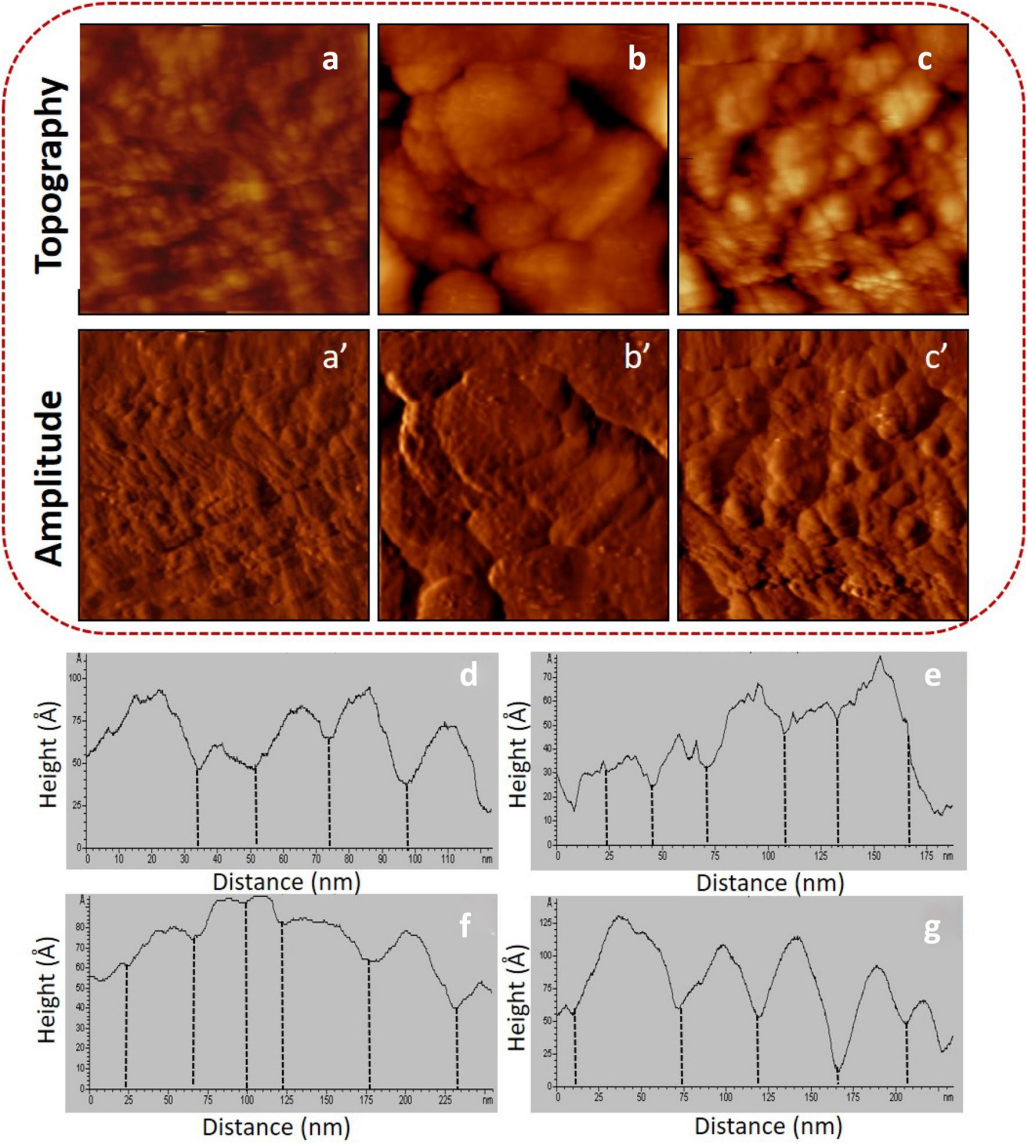
**a**-**c** AFM topography and **a**′–**c**′ amplitude images (image size: 3 × 3 μm^2^) of the wild type cell wall **d**-**g** representative height profiles of the wild type cell wall

**Fig. 2:**
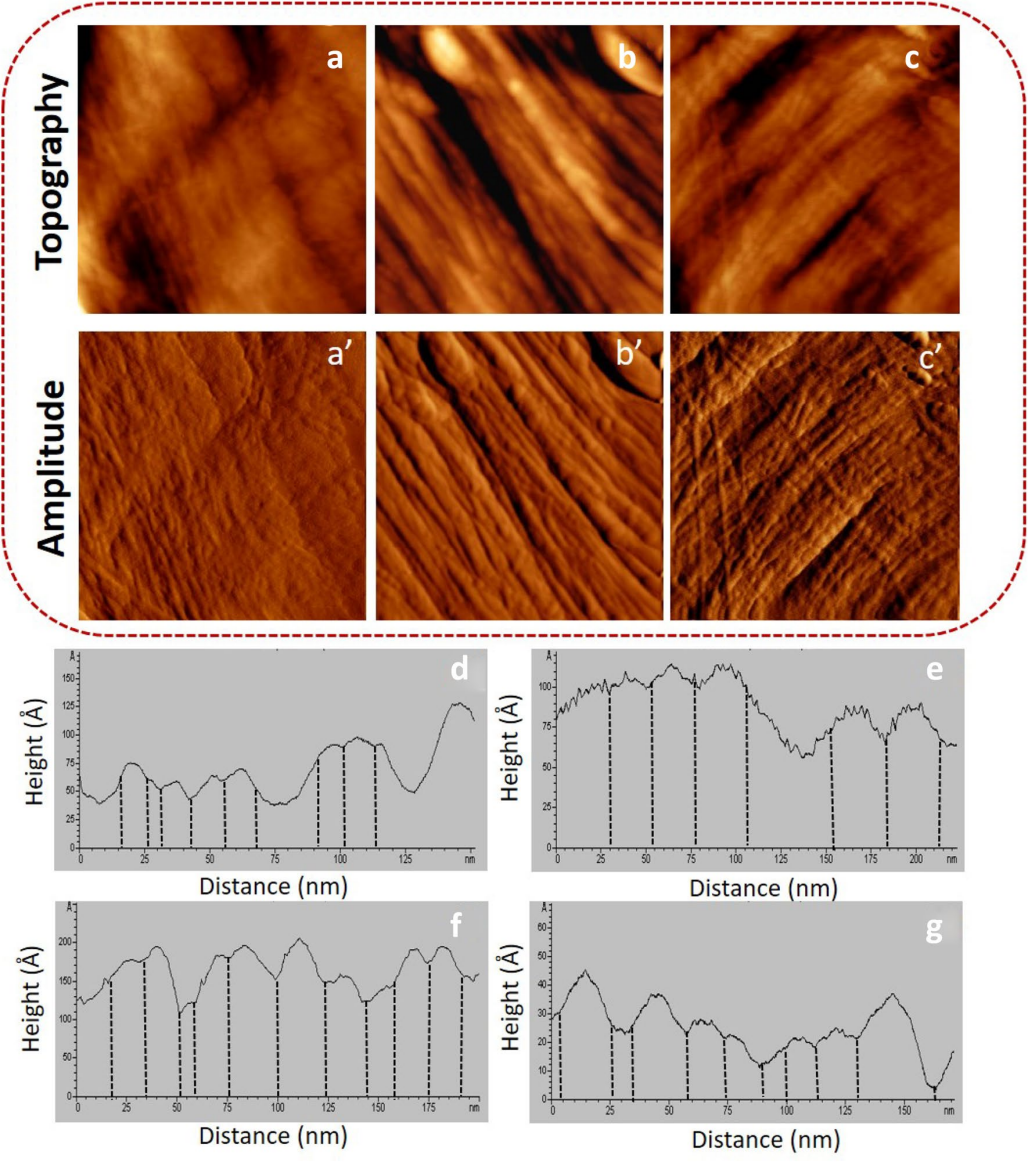
**a**-**c** AFM topography and **a**′–**c**′ amplitude images (image size: 3 × 3 μm^2^) of mutant cell wall. **d**-**g** representative height profiles of mutant cell wall

Because lignin limits the use of biomass for fiber and energy production, strategies for its downregulation are of considerable interest [5]. In this study, the lignin biosynthetic pathway gene expression in the mutant sample was downregulated. The changes in the morphology of *Populus* after genetic pretreatment were investigated by using AFM. Figure 1a–c, a′–c′) shows the AFM topographic and amplitude images of the wild type cell wall. The lignin structures are highly ordered in-between the cellulose and are freely distributed within the wild type cell wall. The images of the mutant cell wall (Fig. 2) show a fibrous network and less irregular remaining structures. Figure 1a–c indicates that lignin with an orientation along with the direction of the cellulose aggregates and fiber structures can almost not be found in Fig. 2a–c. Comparing the structural changes between wild type and mutant cell wall substrates showed a sequence of pretreatment-induced deconstruction, including removal of lignin and increased exposure of cellulose, thus enhancing enzymatic access to cellulose and further biodegradation. It must also be concluded that it is likely that all biomass polymers are orthotropic in nature. Magnified topography images of both wild type and mutant cell wall corroborate this conclusion. Representative zoom-in images are shown in Additional file 1: Figs. S2 and S3, respectively. The cross-section analysis along the green lines is presented in Figs. 1d–g and 2d–g. The height profiles in Fig. 1d–g show that the widths of aggregated structures within wild type cell walls range within 30-55 nm. For the mutant cell wall, the bunched structure widths all remain below 25 nm (Fig. 2d–g. Then, each topography image was equally divided into eight parts equally and their roughness was statistically evaluated. The average roughness value was determined by the equation below:

$$ R_{q} = \sqrt {\sum {(z_{i} - z_{ave} )^{2} /N} } $$

where *z*_*i*_ represents the value of *z* at a particular point, *z*_*ave*_ represents the average value of *z*, and N represents the number of points within a range.

**Fig. 3:**
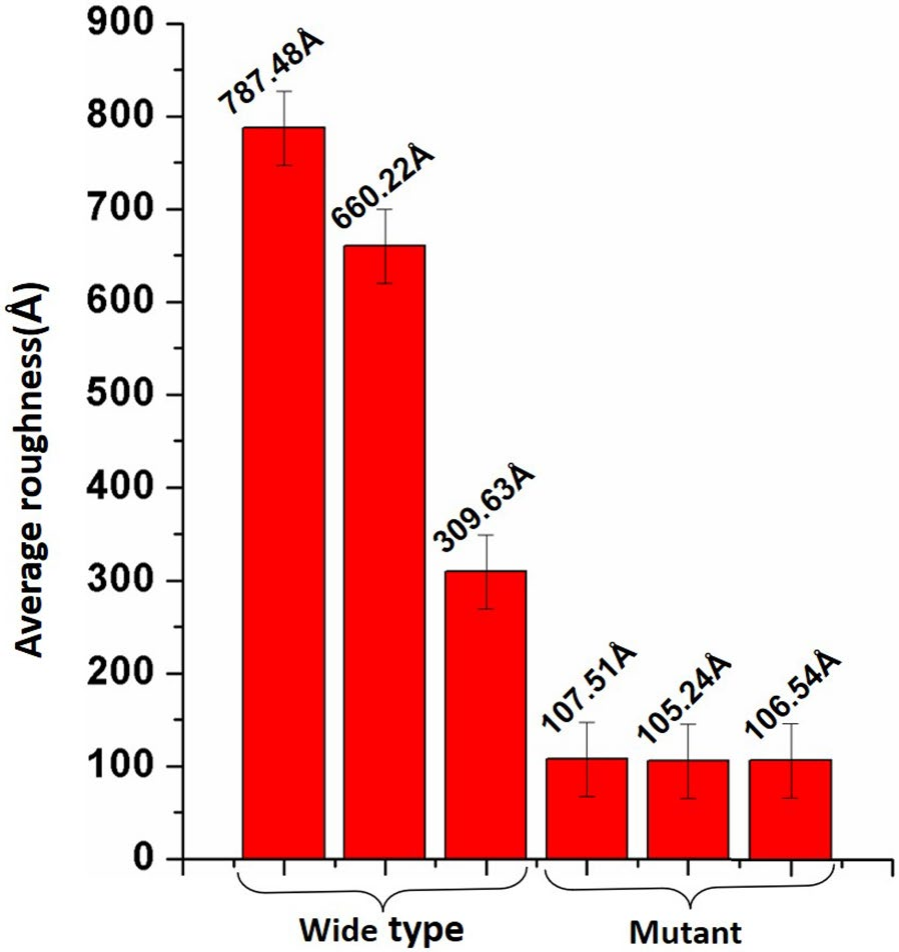
Average surface roughness of wild type and mutant cell walls. *Error bars* indicate standard deviation

Figure 3 shows the average roughness of each picture. The average roughness of the mutant cell wall is approximately 10 nm. The average roughness of the wild type cell wall are 78, 60, and 30 nm which is much higher than that of the mutant cell wall. Furthermore, the surface of mutant cell wall with a much lower and consistent roughness is much flatter than the wide type. These data further confirm that genetic pretreatment effectively inhibited the production of an irregular lignin structure, and the mutant biomass of more exposed fiber structures can be more easily biodegraded [30].

Pretreatment technologies and the products of biomass have been widely studied, but the detailed enzymatic hydrolysis process of pretreated biomass still remains largely unclear [31]. Therefore, this study employed AFM topographic and recognition imaging to follow the enzymatic hydrolysis process of the mutant cell wall to assess the degrading mechanism at a single molecule level.

### Real-time AFM imaging of mutant *Populus* cell wall hydrolyzed by EG

**Fig. 4:**
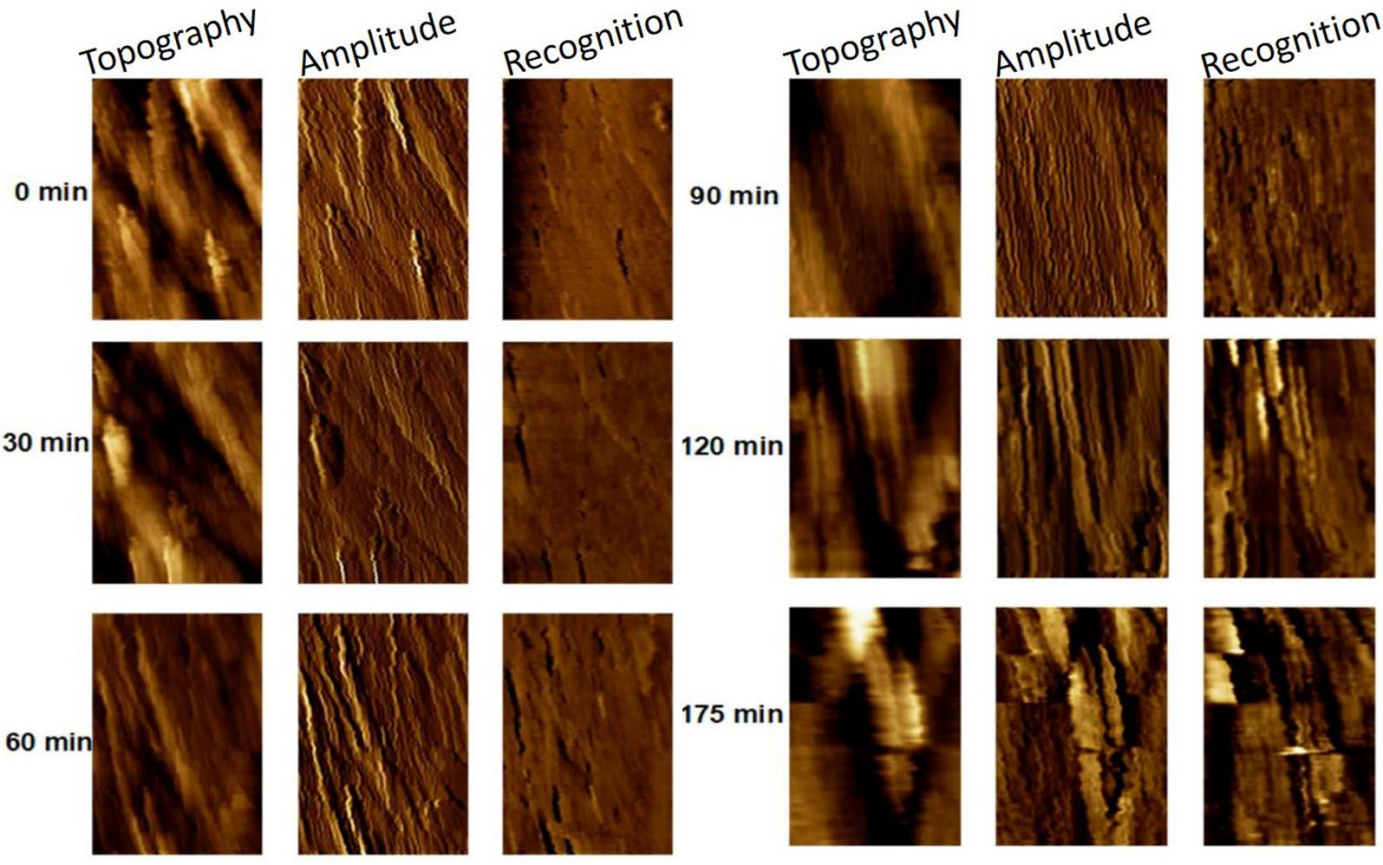
Time-course AFM topography, amplitude, and recognition images of mutant *Populus* cell walls, hydrolyzed by EG (image size: 2 × 1.2 μm^2^). Images show cell wall structures before enzyme hydrolysis and at 30, 60, 90, 120, and 175 min after addition of EG

**Fig. 5:**
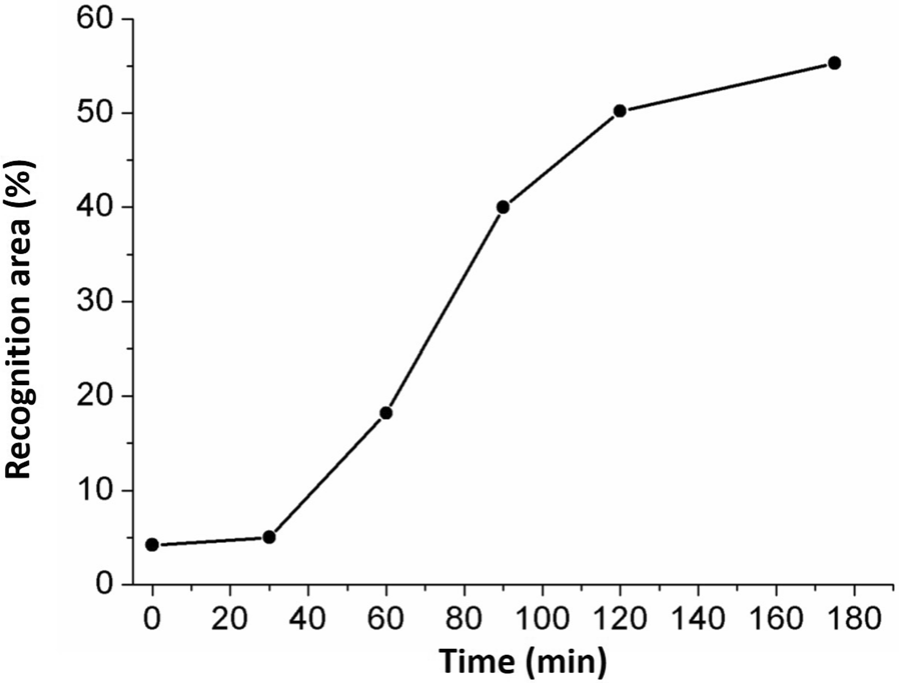
Time course of the recognition area percentage (RAP) from 0 min to 180 min

Analyzing the enzymatic hydrolysis process of mutant biomass enables a deeper understanding of the detailed digestion mechanism of cellulase and can visualize structural changes of the mutant cell wall. Figure 4 focused on an area of 2 × 1.2 μm^2^ in size and shows a series of time-course topography images, amplitude images, and corresponding recognition images of the target mutant cell wall after addition of 0.05 mU EG into the AFM liquid cell. The dark spots in the recognition image represent the crystalline cellulose that exists in the cell wall. Recognition area percentage (RAP) is a measure of percentage of the exposed crystalline cellulose over the whole imaged area. The method has been introduced in the supporting file of a previous publication [28]. Figure 5 shows RAPs changes measured under hydrolysis of EG. The image collected before EG addition (0 min) shows the cell wall constitutes with less recognition signal. The RAP is about 5% at 0 and 30 min. Therefore, the rest of the structures without recognition signal might be amorphous polymers and lignin. It can also be concluded that the amorphous wood polymers play a more dominant role for the longitudinal fiber properties. The next five sets of images in Fig. 4 were collected at different time points during the 175 min of the experiment after injecting of EG solution. During the first 30 min, the recognition signal changed little and few irregular structures disappeared. Most of the EG molecules were likely merely bound on the surface of cellulose to be ready for the following enzymatic activities. Furthermore, the recognition signal first appeared near the place where irregular substrates were located before. Apparently, EG molecules first biodegraded amorphous domains under the irregular substrates; therefore, these substrates detached from the surface and the crystalline domain emerged. Therefore, it can be concluded that a structure with prominent shape is far easier attached by enzyme molecules. In the following steps, EG molecules began to hydrolyze the remaining large area of the amorphous polymer. An increasing number of regular fibers with strong recognition signal appears on the images from 60 min to 90 min. The time-dependent RAP study further indicates that the amorphous domain of cellulose was degraded by EG to expose more of the crystalline domain, leading to more recognition signal. By 120 min, the RAP value is approximately 50%. Then, this value remained stable for the entire imaging time of about 200 min. The process of amorphous hydrolyzation by EG only took 120 min. The amplitude image at 90 min clearly shows successive and intact crystalline cellulose structures. As the experiment progressed, these fiber structures were destroyed. As evidenced in Fig. 4, most of the fibers were distorted and discontinuous from 120 min to 173 min. This means that EG molecules not only degrade amorphous domains but also destroy the crystalline domains of cellulose fibers. Additional file 1: Fig. S4 focuses on one smaller cell wall area before and after conversion. The EG enzyme effectively degraded the amorphous domains (highlighted by red boxes in Additional file 1: Fig. S4a) that cover the crystalline fibers. From the topographic and recognition images after hydrolysis (Additional file 1: Fig. S4b′, 4b′′), the crystalline fibers were shortened to single pieces (highlighted by white lines) with cracks on their edge.

Apparently, EG preferentially hydrolyzed the external amorphous cellulose that covers the crystalline cellulose and then destroys crystalline cellulose fiber structures. Therefore, the action of EG would expose pure crystalline cellulose with more cracks on the surface to be more easily hydrolyzed by CBHI. The recognition signal generating mechanism of the cell wall in the presence of EG is presented in Fig. S5. Because of the covered amorphous domains, the CBM 3a molecule cannot interact directly with crystalline domains. No recognition signal can be observed in the recognition image prior to the injection of EG enzyme solution. When the amorphous domain is removed by EG, an abundant of dark recognition signal emerges in the image. The next step employed AFM imaging to study the degradation mechanism of enzyme CBHI on the mutant cell wall surface.

### Real-time AFM imaging of mutant *Populus* cell walls hydrolyzed by CBHI

The CBHI enzymatic process on extractive cellulose has been reported before [27]. CBHI molecules used the cracks on the surface of the cellulose as starting points and acted in the cellulose elongation direction. This research presents the pictures of how the CBHI molecules work on the surface of the mutant cell wall. In the following, a surface of mutant cell wall was mapped and its morphology was studied. Furthermore, further analysis was performed that combined both the height change and surface roughness change along with time.

**Fig. 6:**
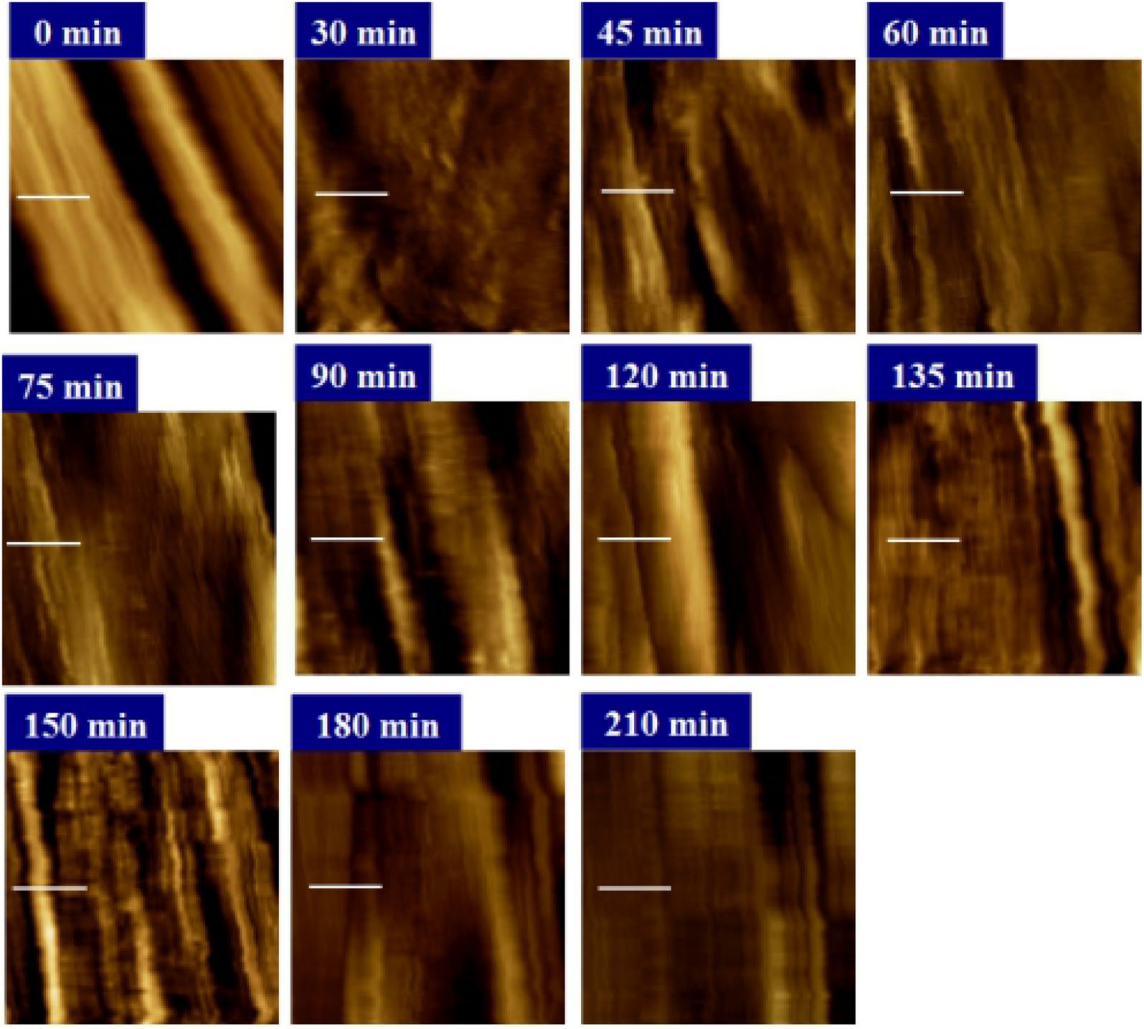
Real-time observation of mutant cell wall incubated with CBHI for 0, 30, 45, 60, 75, 90, 120, 135, 150, 180, and 210 min during the enzymatic hydrolysis process. All topographic images were taken at 3.5 × 3.5 *μ*m^2^ scan size

**Fig. 7:**
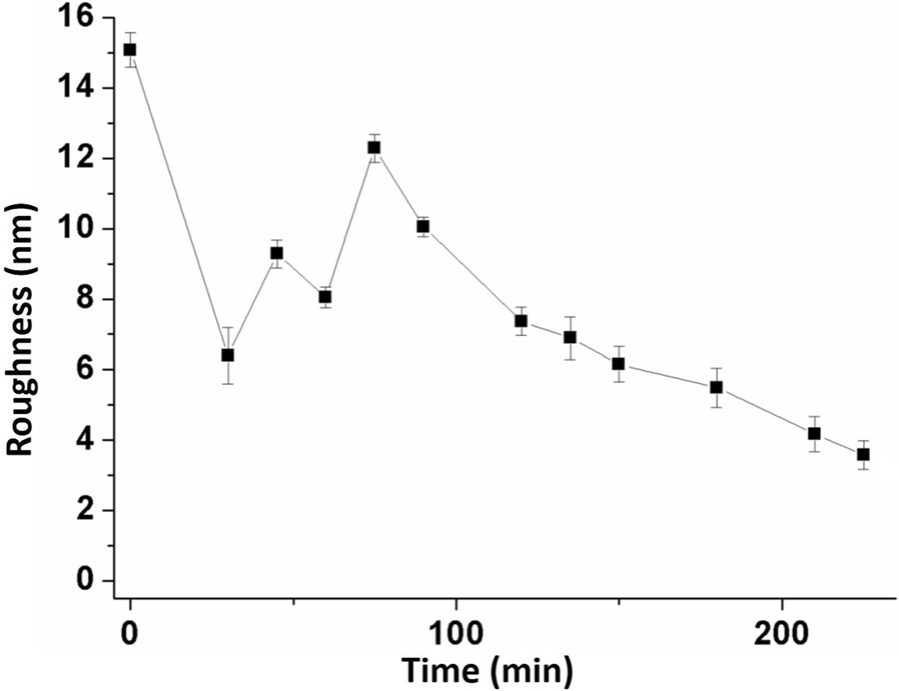
Average surface roughness at each time point. *Error bars* indicate standard deviations

Figure 6 illustrates the progression of enzyme action on the mutant cell wall surface observed over a period of ~ 210 min. After a picture of the cell wall surface was captured, a mixture of CBHI and *β*-G of concentration 0.0001 U was injected into the AFM liquid cell without disengaging the set point of the AFM tip. Images at 30, 45, 60, 75, 90, 120, 135, 150, 180, and 210 min indicate the changes of the cell wall during the reaction after enzyme injection. After 210 min, the cell wall surface became relatively flat and changed little during the following period. Figure 7 shows the average roughness change of the cell wall surface during the whole hydrolysis process. The average roughness of the cell wall surface decreased to about 3 nm at 210 min, where it remained for a period. To analyze cell wall structures in particular, cross-section profiles (Fig. S6) were studied along the white lines marked on AFM topographic images. During the first 30 min, the surface underwent a notable change. The height of the structure along the white line changed from about 300 nm before hydrolysis to about 60 nm at 30 min. Therefore, profile information was only presented from 30 min in Fig. S6 to enable convenient comparison. The middle of this structure is obviously much higher. More molecules could adhere to higher structures with more exposed endpoints as shown in Fig. 6. Therefore, this part biodegraded very fast. During the first 60 min, the higher structures were degraded in order. However, the height suddenly increased to a much higher value at 75 min. Then, the height decreased again. Over the following minutes, the height increased gradually and reached the maximum value at 180 min. At 210 min, the height profile became quite low and flat. Figure 7 shows that the average roughness had an extremum at 75 min. In summary, this phenomenon can be concluded to be similar to the “traffic jam” that Igarashi et al. proposed [32]. In the process of cell wall hydrolysis, many enzymes finished the hydrolysis work on the rear of the cell wall and continued to be attached to the surface. This led to a stack of large numbers of molecules in one place. The resulting blockage could be cleared after the molecules started to move again. As a consequence, glycan chains on the surface of the cell wall peeled off. This peeling process resulted in an increase in the cell wall height. Then, parts of cell wall structures were removed by this peeling. The cell wall surface exhibited a new morphology after the peeling. Two possible phenomena could be responsible: (1) an uneven surface was generated and the peeling happened again; (2) the surface became fairly flat, and layer-by-layer hydrolysis proceeded in order. Obviously, the cell wall shown in Fig. 4 finished the first peeling within 90 min and generated another peeling at 180 min. The flat cell wall surface produced in 210 min did not generate any further enzyme molecules stacks. The average roughness of the cell wall achieved almost 12 nm within 75 min then decreased to about 3 nm in the end (Fig. 7). Therefore, most of the cell wall structures had only one peeling at 75 min.

**Fig. 8:**
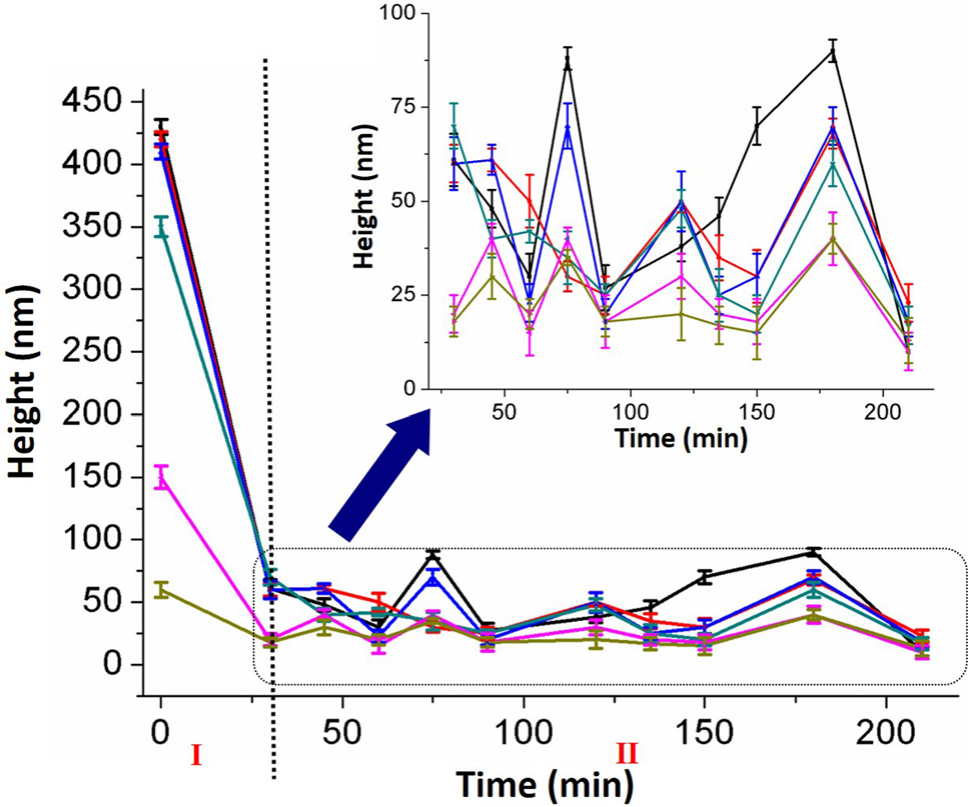
Statistic of different generated peak heights during the whole hydrolysis process. Eight cell wall structures for one hydrolysis process curve. Height changes were divided into two parts. The second part was amplified at the upper right corner. *Error bars* indicate standard deviations

Considering that the hydrolysis mode may be related to the height of the substrate, several structures of different heights on this cell wall surface were chosen and their height information during the whole hydrolysis process was collected. These data are exhibited in Fig. 8. All structures were biodegraded markedly within the first 30 min. However, their height change in the following time was not statistically significant. This suggests that the occurred peeling was more closely related to the cell wall roughness rather than its height. In other words, when the height difference between structures is sufficiently large, enzymes can easily be stacked. Keeping the cell wall roughness below 3 nm can avoid such peeling, and then, the cell wall can progressively be depolymerized to soluble sugars without stopping the movement of enzymes. Above all, to achieve a more efficient hydrolysis, the mutant cell wall can be preprocessed to get a flatter surface and then, biomass can be effectively depolymerized.

## Conclusions

By using AFM topography and recognition imaging, this study visually compared the ultrastructure of wild type and mutant cell walls, and then conducted real-time visualization to follow the details of the hydrolysis process of mutant cell walls. Effective removal of lignin and increased exposure of cellulose were identified in the mutant cell wall, which enhanced the access of enzymes to cellulose and thus improved biodegradation. EG preferentially hydrolyzed external amorphous polymers that covered the surface and then destroyed crystalline structures. Therefore, the action of EG would expose pure crystalline cellulose with a low degree of crystallinity to be more easily hydrolyzed by CBHI. The visualization of the CBHI enzymatic hydrolysis process revealed that the overall digestibility actively correlated with the roughness of the cell wall surface. A cell wall with a roughness less than 3 nm can be progressively depolymerized to soluble sugars with less peeling. Based on the detailed digestion process of mutant cell walls pretreated by genetic engineering, the future research with the aim to enhance biomass hydrolysis efficiency will primarily focus on combining different pretreatment technologies. Genetic pretreatment can effectively inhibit irregular lignin structure production within the cell wall. Selecting a collaborative method (such as milling, extrusion, and microwaving) to ensure a cell wall surface roughness below 3 nm will be of great value.

## Supplementary information

**Additional file 1.** Additional figures and tables.

## Data Availability

Not applicable.

## Abbreviations

FTIR: Fourier Transform Infrared
NMR: Nuclear Magnetic Resonance
SEM: Scanning Electron Microscope
GFP: Green fluorescence protein
AFM: Atomic force microscopy
HS-AFM: High-speed atomic force microscopy
EG: Endoglucanases
CBHI: Cellobiohydrolase I
*β*-G: *β*-Glucosidases
CBM: Carbohydrate-binding module
RAP: Recognition area percentage
MAC: Magnetic AC mode
