# Supplementary material for: Plant cell wall hydrolysis process reveals structure–activity relationships
Source: Plant Methods. 2020 Nov 3;16:147. doi: 10.1186/s13007-020-00691-5 (PMC7640438; doi:10.1186/s13007-020-00691-5)
Supplement: Supplementary file 1 — Additional file 1. Additional figures and tables. [file 13007_2020_691_MOESM1_ESM.docx]

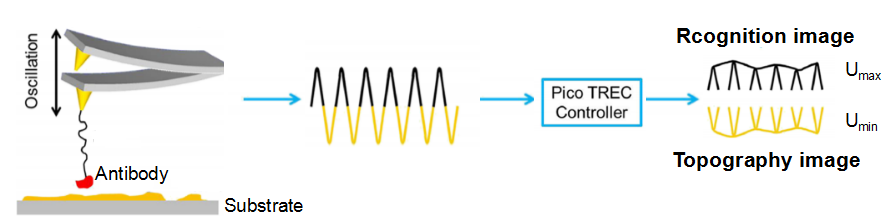


**Fig. S1** Schematic of AFM recognition imaging. Topography and recognition

signal division by PicoTREC controller.

The method is based on detecting small shift in the peak value of the cantilever deflection signal that occur when a tip-tethered molecule (Antibody) binds to the target antigen on the sample substrate surface. A site-directed cross-linker may be desired to orient the attached antibody molecule, and allow the antibody molecule to freely diffuse within a certain volume. Topography and recognition images are simultaneously obtained using by PicoTREC Controller. Maxima (U_max_) and minima (U_min_) of each sinusoidal cantilever deflection period are depicted and fed into the AFM controller, with U_min_ driving the feedback loop to record the topography image and U_max_ providing the data for construction of the recognition image.

Table S1 Overview of biomass visualization techniques

| **Techniques** | **Schematic setup** | **characteristics** | **Application** | **References** |
| --- | --- | --- | --- | --- |
| Confocal Laser Microscopy (CLSM) | 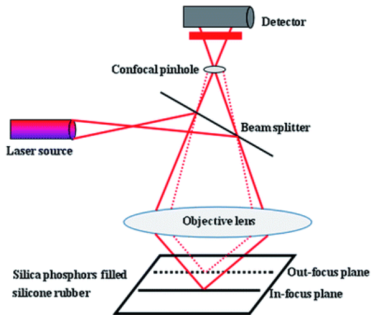 | - Fluorescense quenching - Rapid analysis - Difficult to obtain the reproducible apectra | - Biochemical imaging - Rapid identification of microorganisms | [1]  [2] |
| Transmission Electron Microscopy (TEM)  3D-TEM | 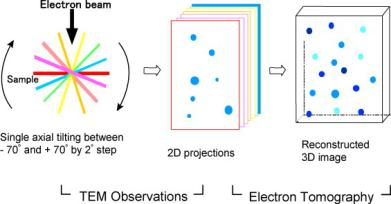 | - Easy operation - Simple quantitative analysis | - Biochemical imaging - Remodeling of light-harvesting complexes - Volumetric analysis | [3] |
| Raman Microscopy (RM） | 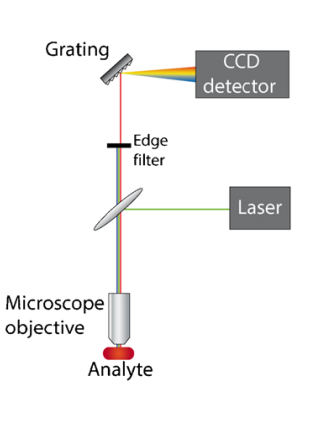 | - Molecular/chemical fingerprintig - Water insensitivity - Spatial resolution in the μm-range - No staining required - Fluorescence interference - Long analysis time - Limited sensitivity | - Biochemical imaging - Cell sorting - Identification of single cells | [4] |
| AFM  HS-AFM | 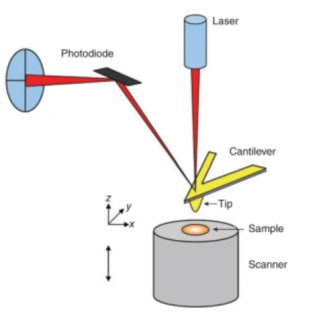 | - High sensitivity - Specific identification - Label -free of the sample - The tip is easy to pollute - Background noise | - Biochemical imaging - High-resolution measurement of the architecture of cell walls - Analysis of dynamics on cell surface - Non-invasive cell tracking - Quantitative Imaging - *In-situ* imaging | [5]  [6]  [7] |

[1] Liu M, Kang M, Mou Y, Chen K, Sun R: Visualization of filler network in silicone rubber with confocal laser-scanning microscopy. *RSC advances,* 2017, 7.84: 53578-53586.

[2] Dong M, Wang S, Xu F, Wang J, Yang N, Li Q, Chen J, and Li W: Pretreatment of sweet sorghum straw and its enzymatic digestion: insight into the structural changes and visualization of hydrolysis process. *Biotechnology for biofuels,* 2019, 12: 1-11.

[3] Shuhei O, *et al.*: Highly efficient lipid production in the green alga Parachlorella kessleri: draft genome and transcriptome endorsed by whole-cell 3D ultrastructure.*Biotechnology for biofuels*, 2016, 9.1: 13.

[4] Ivleva N P, Kubryk P, Niessner R: Raman microspectroscopy, surface-enhanced Raman scattering microspectroscopy, and stable-isotope Raman microspectroscopy for biofilm characterization. *Analytical and bioanalytical chemistry*, 2017, 409.18: 4353-4375.

[5] Hinterdorfer P, Dufrene Y F: Detection and localization of single molecular recognition events using atomic force microscopy. *Nature methods*, 2006, 3.5: 347-355.

[6] Zhang Y, Zhang M, Reese R A, Zhang H, and Xu B: Real-time single molecular study of a pretreated cellulose hydrolysis mode and individual enzyme movement. *Biotechnology for biofuels,* 2016, 9: 85.

[7] Casdorff K, Tobias K, Ingo B: Nano-mechanical characterization of the wood cell wall by AFM studies: comparison between AC-and QI™ mode. *Plant Methods*, 2017, 13.1: 1-9.


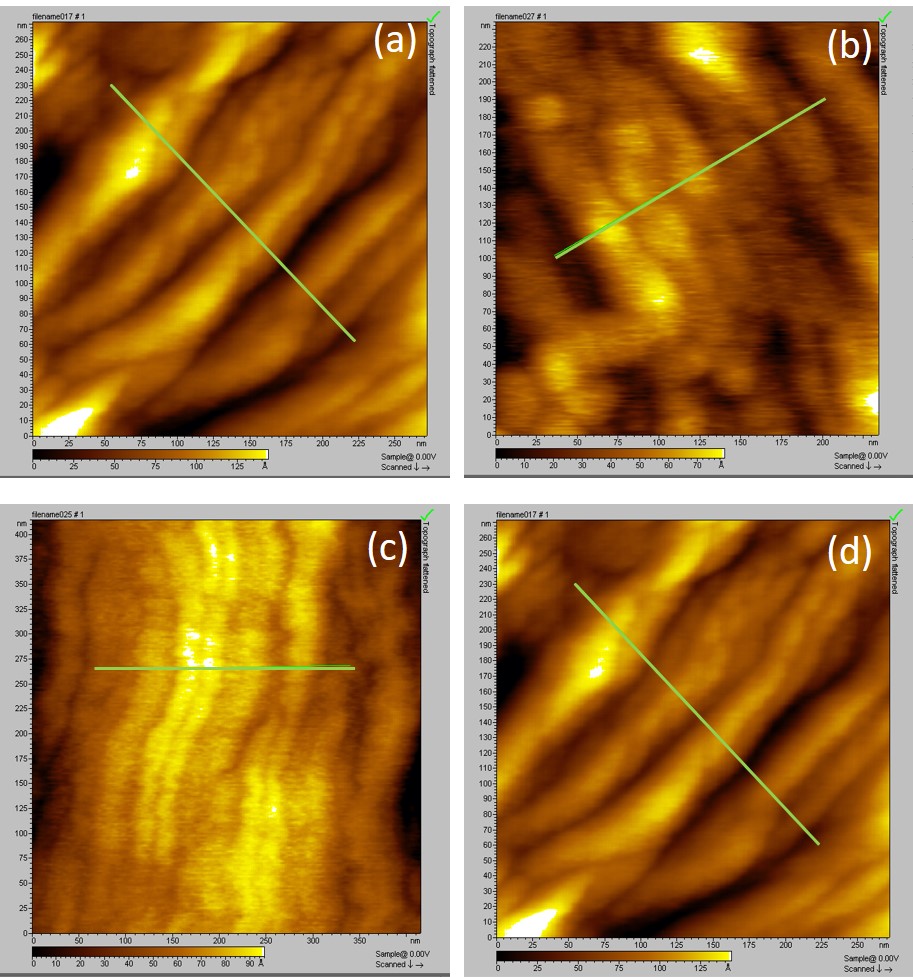


**Fig. S2** Zoom-in topography images of wild type cell wall. Green lines are drawn for the profiles.

Four zoom-in topography images were picked from Fig. 1a-c. The cross-section analysis along the green lines in Fig. S2a-d is presented in Fig. 1d-g.


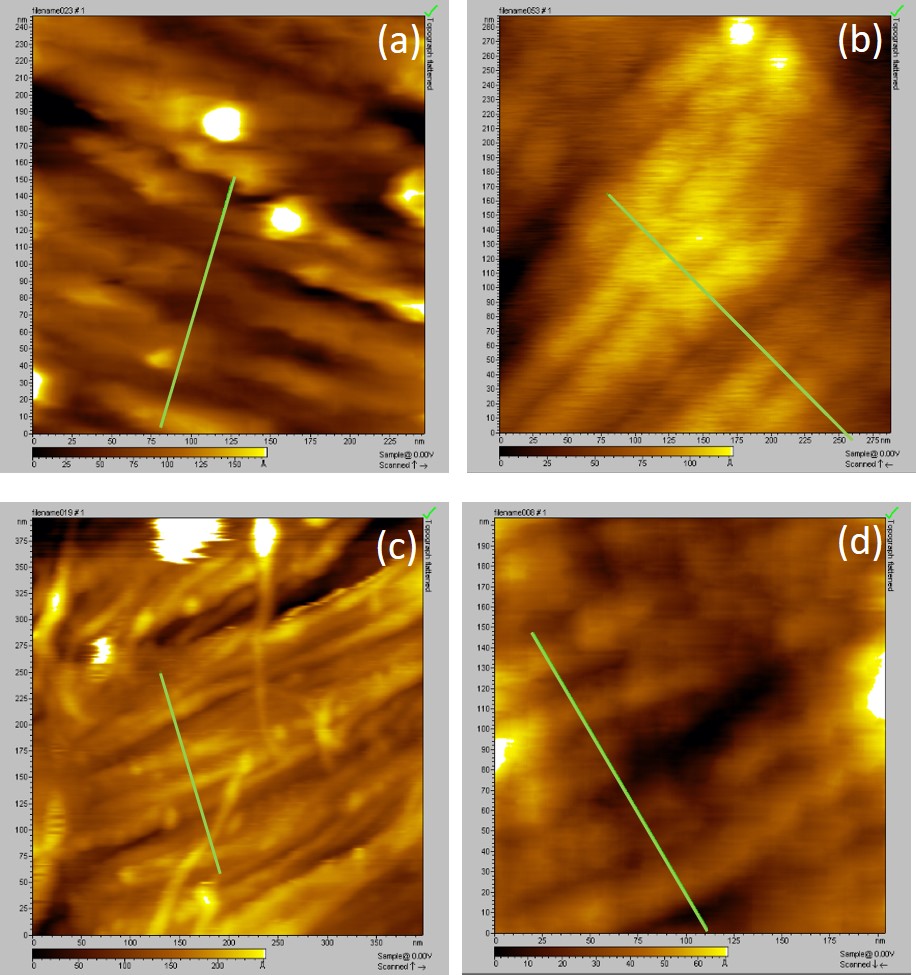


**Fig. S3** Zoom-in topography images of mutant cell wall. Green lines are drawn for the profiles.

Four zoom-in topography images were picked from Fig. 2a-c. The cross-section analysis along the green lines is presented in Fig. 2d-g.


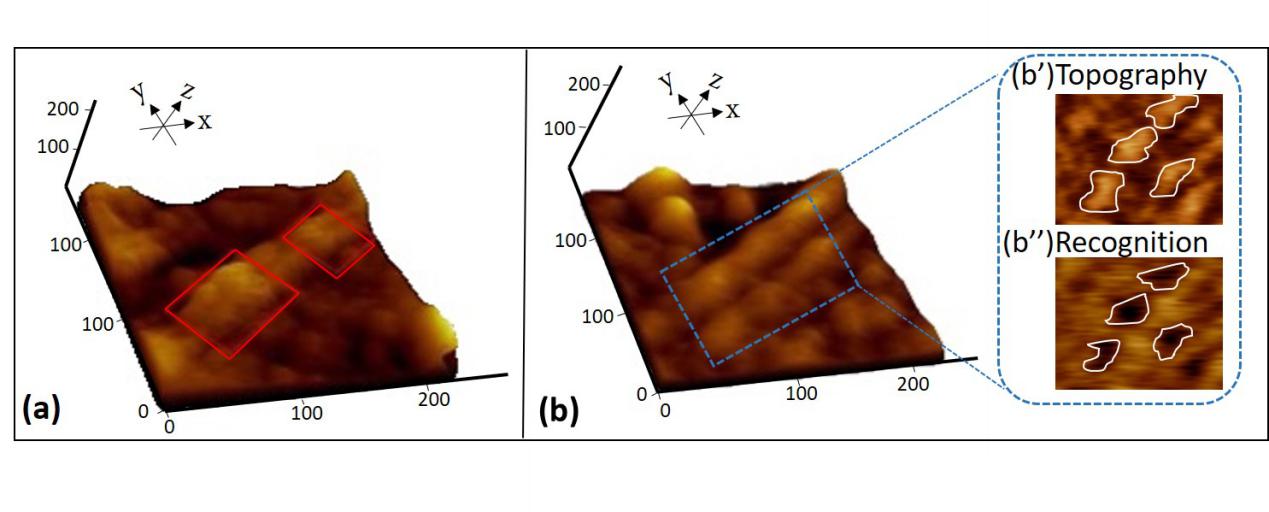
 **Fig. S4** 3D images before and after EG hydrolysis (0.2×0.18 *μ*m^2^). b’ and b’’ are respectively the zoom-in topography and recognition images (60×50 nm^2^) after hydrolysis is complete.

Red boxes highlight the amorphous domains removed by EG.


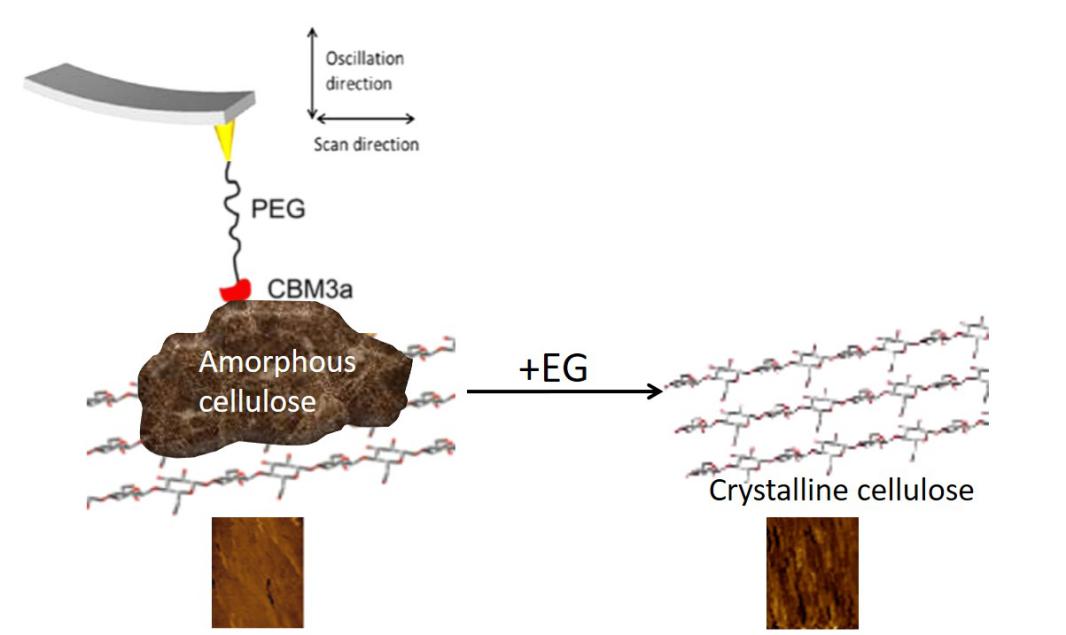


**Fig. S5** Schematic presentation of the recognition signal generating mechanism of cell wall in the presence of EG. After the degradation of amorphous cellulose by EG, recognition signal appears by the direct interaction between the covered crystalline cellulose and CBM 3a molecule.


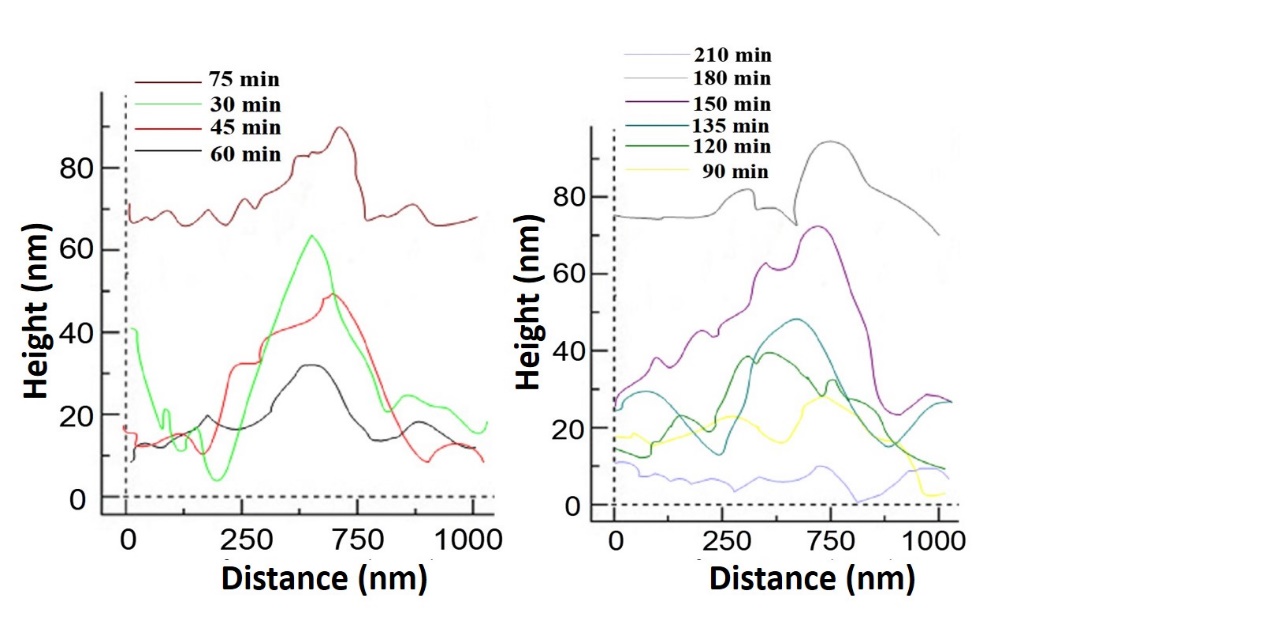


**Fig. S6** Cross-section analysis along the white lines on each topographic image of the mutant cell wall incubated with CBH I.
